# Quinic acid ameliorates ulcerative colitis in rats, through the inhibition of two TLR4‐NF‐κB and NF‐κB‐INOS‐NO signaling pathways

**DOI:** 10.1002/iid3.926

**Published:** 2023-08-08

**Authors:** Maryam Ghasemi‐Dehnoo, Zahra Lorigooini, Hossein Amini‐Khoei, Milad Sabzevary‐Ghahfarokhi, Mahmoud Rafieian‐Kopaei

**Affiliations:** ^1^ Medical Plants Research Center, Basic Health Sciences Institute Shahrekord University of Medical Sciences Shahrekord Iran

**Keywords:** apoptosis, inflammation, oxidative stress, quinic acid, rat, ulcerative colitis

## Abstract

**Objective:**

In this study, the therapeutic effect of quinic acid (QA), which has anti‐inflammatory activity, was investigated on acetic acid‐induced colitis in male Wistar rats.

**Methods:**

Ulcerative colitis (UC) was induced in rats by acetic acid intrarectally, and the protective effects of QA in 10, 30, 60, and 100 mg/kg doses were investigated. Rats were treated for 5 days and their colon tissues were dissected out at the end. Macroscopic and histopathological examinations were performed in colon tissues. Also, the expression of inflammatory and apoptotic genes, including TLR4, IL‐1β, INOS, IL‐6, TNF‐α, NF‐κB, Caspase‐3, Caspase‐8, Bax, and Bcl‐2, was measured. Biochemistry indices, such as malondialdehyde (MDA) and nitrite oxide (NO) content, in addition to, total antioxidant capacity (TAC), superoxide dismutase (SOD), catalase (CAT), and enzymes activities were also assessed.

**Results:**

Colitis increased the levels of MDA and NO, and enhanced the inflammatory and apoptotic gene expressions, while reducing the SOD and CAT enzymes activity, and TAC levels in the colitis rats. Also, results showed that colitis was associated with the infiltration of inflammatory cells, epithelium damage, and edema in colon tissue. QA significantly ameliorated histopathological indices, oxidative stress, inflammation, and apoptosis in colitis rats.

**Conclusion:**

QA ameliorated UC through the inhibition of two TLR4‐NF‐κB and NF‐κB‐INOS‐NO signaling pathways, which results in the reduction of colitis complications, including oxidative stress, inflammation, apoptosis and histopathological injuries in rats. Therefore it can be concluded, that QA exerts its therapeutic effects through antiapoptotic, antioxidant, and anti‐inflammatory properties.

## Graphical abstract

Quinic acid ameliorated ulcerative colitis through the inhibition of two TLR4‐NF‐κB and NF‐κB‐INOS‐NO signaling pathways, which results in the reduction of colitis complications, including oxidative stress, inflammation, apoptosis, and histopathological injuries in rats.

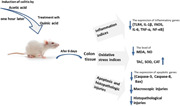

## 1 INTRODUCTION

Inflammatory bowel disease (IBD) is a chronic idiopathic inflammatory complication of the gastrointestinal tract, and its underlying cause is still unknown.^1^ Ulcerative colitis (UC) and Crohn's disease (CD) are the two main types of IBD.^2^ UC covers the colon and rectum, while CD can involve the entire length of the gastrointestinal tract.^3^ The pathogenesis of IBD is complex. IBD affects various components of the mucosal immune system, including intestinal epithelial cells, innate immune cells, adaptive immune cells, and their secreted mediators, including eicosanoids and cytokines.^4^ Accumulation of these mediators, cells, and neutrophil infiltration may cause severe inflammation of the intestine. Toll‐like receptor 4 (TLR4) signaling pathway may play a crucial role in intestinal inflammation in UC. Activation of this pathway leads to the activation of NF‐κB signaling and subsequent production of cytokines such as IFN‐γ, IL‐1β, TNF‐α, IL‐6, IL‐8, and IL‐12.^5^ On the other hand, studies show that flavonoids and polyphenols can inhibit NF‐κB signaling through interaction with TLR4.^6^ As a result, these natural compounds are effective in reducing inflammation caused by UC,^6^, ^7^ also inhibit UC‐induced oxidative stress by inhibiting the production of free radicals and reducing lipid peroxidation products.^8^ In this regard, the effects of these compounds have also been proven to increase antioxidant enzyme levels, including GSH and SOD.^9^ Standard treatments for UC, include anti‐inflammatory and immunosuppressive drugs, and in some cases surgical procedures. But the common treatments are certainly not effective in all cases and have many side effects.^10^ Researchers are still trying to find effective and safe drugs. Studies in recent years have shown that plant compounds with anti‐inflammatory and antioxidant properties have been very useful and promising in this field.^11^ Quinic acid, tetrahydroxycyclohexanecarboxylic acid (QA), is a carboxylic acid cyclohexane with polyphenolic structure found in the bark of the cinchona, coffee beans,^12^ and other plants, including sweet potatoes, apples, and peaches.^13^ It has the radioprotective,^14^ antidiabetic,^15^ anti‐neuroinflammatory, and antioxidant activities.^12^, ^16^

The present study aimed to determine the effect of QA in prevention and improvement of rats with UC induced by acetic acid, considering its possible antiapoptotic, antioxidant, and anti‐inflammatory properties.

**Figure 1:**
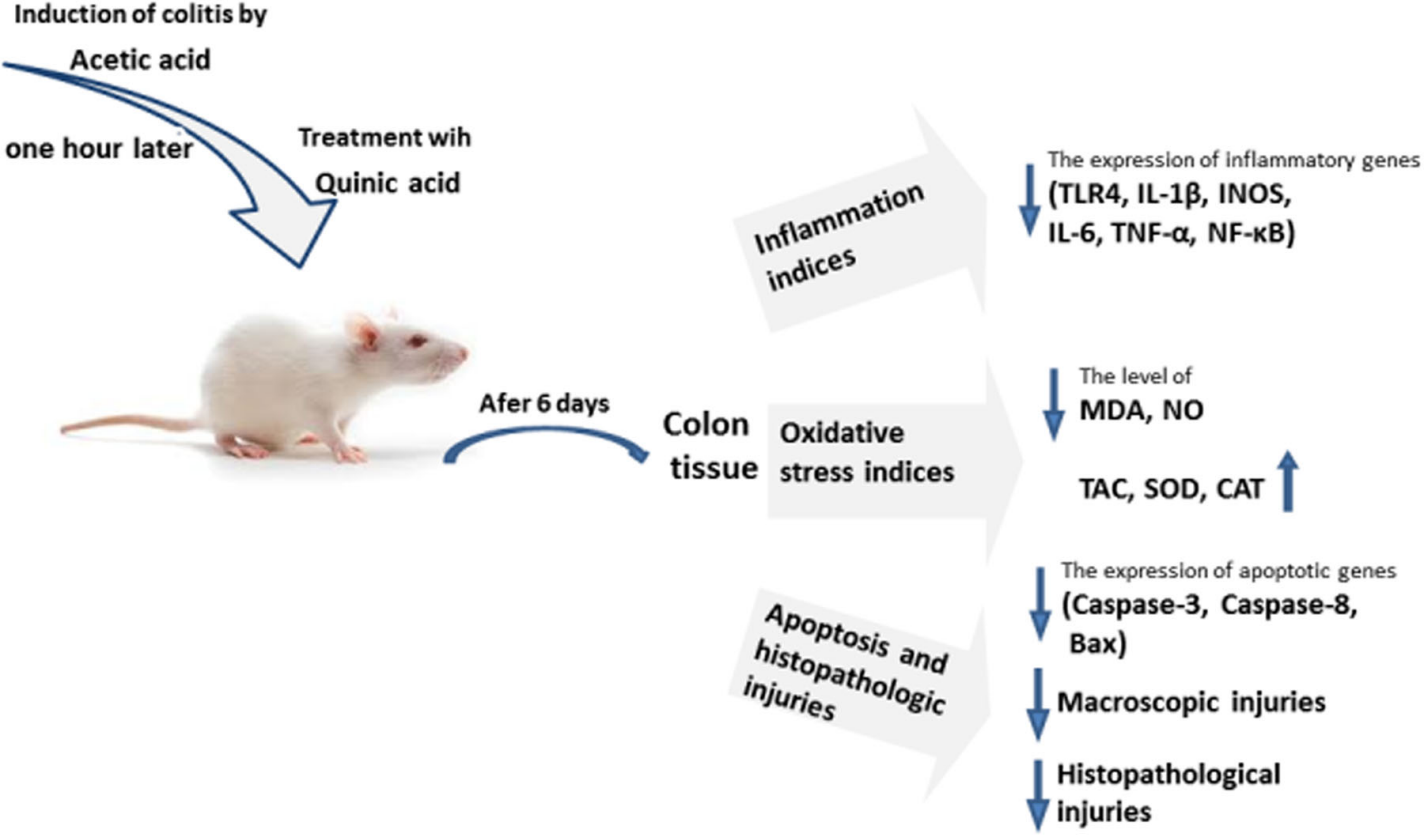
Graphical abstract of the manuscript.

The graphical abstract of the manuscript has been presented in Figure 1.

## 2 MATERIAL AND METHODS

### 2.1 Animals and drugs

Fifty‐six healthy male Wistar rats weighing between 225 and 240 g were used in the present study (Pasteur Institute). Animal exclusion criteria were animal death, disease, or infection during the study.

All the rats were kept in cage; water and standard laboratory pellets were available ad libitum. Conditions for keeping rats included: temperature 20–22°C, relative humidity 40%–60%, and 12 h light–dark cycles.

This protocol was approved by the Ethics Committee of the university (IR.SKUMS.REC.1399.113). All animal handling procedures during the study were performed according to the rules of the National Academies Press (US), Guide For The Care and Use of Laboratory Animals. 8th ed.

All possible efforts were made to improve animal welfare and minimize the use of animals in the study.

QA with a purity of 98% HPC was obtained from Sigma‐Aldrich Company (Product no. 138622).

### 2.2 Study design

The main supervisor of the study, considered indicators to identify the different experimental groups. According to this, none of the people who participated in the different stages of animal treatment, collecting the results, and data analysis were aware of the allocation of the groups.

In this study, 56 rats were randomly divided into seven groups (*n* = 8):

Group I: Control group (Intact rats receiving 2 mg/kg/day phosphate‐buffered saline [PBS], instead of medicine).
Group II: Colitis group (Colitis rats receiving 2 mg/kg/day PBS, instead of medicine).
Groups III–VII: Treatment groups (Colitis rats receiving 10, 30, 60, 100 mg/kg QA, and 2 mg/kg dexamethasone as medicine) by gavage for 5 days, 1 h after inducing colitis.

Groups intended for induction of colitis were fasting for 1 day, then received acetic acid (0.8 ml 7%, intrarectally at 8 cm near the anus) for 30 s. Rats in the control group received similarly 0.8 mL PBS.^17^

### 2.3 Evaluation of macroscopic scores

At the end of the study, rats in all groups were euthanized following anesthetization by xylazine (13 mg/kg) and ketamine (87 mg/kg). The rat's colon was removed and opened lengthwise. Then, after rinsing with saline, ocular observation was performed to evaluate the degree of inflammation of the colon. Macroscopic lesions in the tissues were recorded for the colon of each rat by the Wallace and Keenan scoring system, based on the absence or presence of ulcer and the area of inflammation.^18^ Using this semi‐quantitative scoring system, the macroscopic damage was classified into the following five categories; 0: no inflammation and no ulcer 1: localized hyperemia and no lesion, 2: lesions without any hyperemia, 3: lesions with only one area inflammation, 4: inflammation in two or more areas with lesions, 5: lesions larger than 2 cm.^18^

Finally, the colon tissue was kept in appropriate conditions for biochemical, histopathological, and real‐time tests.

### 2.4 Histopathological examination

After 1 week of fixing the prepared colon samples in 10% formaldehyde in PBS, they were dried using graded ethanol. Then, they were placed in paraffin wax and thin pieces were prepared from them. After deparaffinization of the sections using xylene, staining was performed with hematoxylin–eosin (H&E). The sections were evaluated using a light microscope, and morphological changes were recorded. The severity and extent of inflammation, as well as the percent of involvement and crypt damage, were considered as pathological assessment criteria of colitis. Table 1 shows scales defined for each of the criteria.^19^

**Table 1:** Scoring system for pathological assessment of colitis.^18^

Scoring parameter	Score definition
Severity of inflammation	None: 0, Mild: 1, Moderate: 2, Severe: 3
Extent of inflammation	None: 0, Mucosa: 1, Mucosa and submucosa: 2, Transmural: 3
Crypt damage	None: 0, Basal 1/3 damaged: 1, Basal 2/3 damaged: 2, Crypts lost, Surface epithelium present: 3, Crypts lost, Surface epithelium lost: 4
Involvement percent	0%: 0, 1%–25%: 1, 26%–50%: 2, 51%–75%: 3, 76%–100%: 4

### 2.5 Biochemical analysis

To evaluate the biochemical indicators, the colon tissues were first homogenized in buffer containing 1.15% KCI with a ratio of 1/10, then all assessments were performed on homogenized tissue of the colon.^20^

Malondialdehyde (MDA) levels were evaluated by a spectrophotometric method, described by Ohkawa based on reactions with thiobarbituric acid; the result was presented as μg/mL.^21^

CAT activity levels were evaluated based on the H_2_O_2_ decomposition principle at 240 nm; the result was presented as U/mg protein.^22^

SOD activity levels were evaluated based on a previously described method by Mishra and Fridovich, the result was presented as U/mg protein.^23^

Nitric oxide level was evaluated based on sodium nitrate reaction with Griss reagent; the result was presented as μmol/L.^24^

Total antioxidant capacity (TAC) levels in homogenized tissue were evaluated by FRAP assay method, the result was presented as μg/ml.^25^

### 2.6 Real‐time polymerase chain reaction analysis

Total RNA was extracted from the colon tissue, according to the RNA extraction protocol by TRIzol (Invitrogen). The extracted RNA was evaluated by a nanodrop device, and then, was used for cDNA synthesis, using a cDNA synthesis kit (Thermo Scientific RevertAid First Strand cDNA Synthesis Kit). The next step was real‐time polymerase chain reaction (RT‐PCR), which was done by the SYBR Premix Ex Taq technology on a light cycler apparatus (Rotor gene Diagnostics; Takara).

In this study, the thermal cycling program profile was as follows: 95°C for 30 s, 45 cycles of denaturation (at 95°C) for 5 s, annealing step (at 60°C) for 15 s, and extension (at 72°C) for 15 s. Melting curve for each PCR product was checked to ensure that all primers were single product. Normalization of the target gene transcription values was performed using the $2^{‒{\mathrm{\Delta \Delta C}}_{t}}$ relative expression formula, relative to the housekeeping β‐actin gene.^26^

The sequences of primers used for qRT‐PCR are presented in Table 2.

**Table 2:** Primer sequences.

Primer sequence	Reverse	Forward
β‐actin	AGGAAGGAAGGCTGGAAGAGA	AGAGGGAAATCGTGCGTGAC
TLR4	CACAGCAGAAACCCAGATGAAC	AGAGGAAGAACAAGAAGCAACAAC
TNF‐α	CGTGTGTTTCTGAGCATCGTAGT	CTGGCGTGTTCATCCGTTCT
IL‐6	TGTTGTGGGTGGTATCCTCTGT	TGCCTTCTTGGGACTGATGTTG
INOS	GCACACGCAATGATGGGAAC	GAAGAGACGCACAGGCAGA
IL‐1β	AGCAGGTCGTCATCATCCCA	TTCAAATCTCACAGCAGCATCTC
Caspase‐8	CGTAGTGTGAAGATGGGCTGT	CTGACTGGCGTGAACTATGATG
Bax	CAGTTGAAGTTGCCGTCTG	GAGGATGATTGCTGATGTGGATA
Caspase‐3	GCCATATCATCGTCAGTTCCAC	AAGCCGAAACTCTTCATCATTCA
NF‐κB	GTCTTATGGCTGAGGTCTGGTC	CGTGAGGCTGTTTGGTTTGAG
Bcl‐2	ACAGCCAGGAGAAATCAAACAGA	GGAGCGTCAACAGGGAGATG

### 2.7 Statistical analyses

The sample size was calculated by power calculations using G power software (ver.3.1.7, Franz Faul, Universitat Kiel). We set *α* error at .05 and power (1 − *β*) at .8 and the required total sample size per group was calculated as 6–8 in behavioral tests and 3–4 in molecular experiments.

Statistical analysis was performed using one‐way analysis of variance (ANOVA) test, followed by Tukey's post hoc test in GraphPad Software (version 8.4.3). Results are expressed as mean ± standard error mean (SEM). The 95% probability level (*p* < .05) was considered to express significant differences between groups.

## 3 RESULTS

### 3.1 Effects of QA on macroscopic changes

**Figure 2:**
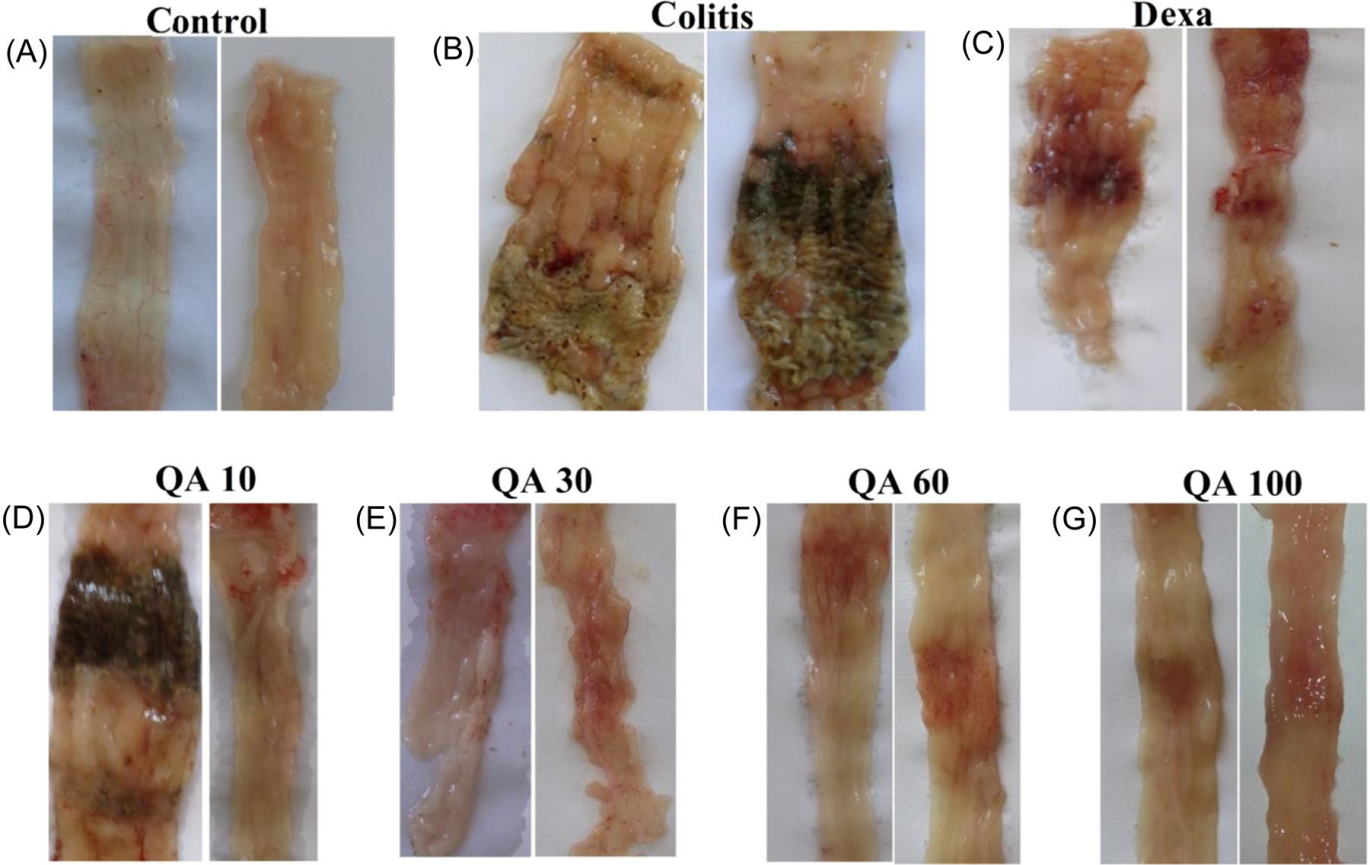
Photographs related to the examination of colon tissue changes through visual observation. Dexa, dexamethasone (2 mg/kg); QA 10, QA 30, QA 60, and QA 100, Quinic acid in doses of 10, 30, 60, and 100 mg/kg.

During ocular observation, severe tissue damage including (inflammation, hyperemia, edema, and ulcer) was observed, in the colon of colitis rats (Figure 2). Scoring of this damage showed a significant increase, in comparison to the control samples (4.444 ± 0.2422, *p* < .001).

Treatment of colitis samples with QA in four concentrations of 10, 30, 60, 100 mg/kg and dexamethasone, resulted in the healing of ulcers and tissue damages at a significant level, respectively: (2.556 ± 0.5031, *p* < .01), (1.556 ± 0.2422, *p* < .001), (1.444 ± 0.2422, *p* < .001), (1.333 ± 0.2357, *p* < .001), and (1.778 ± 0.2778, *p* < .001).

### 3.2 Effects of QA on histopathological changes

**Figure 3:**
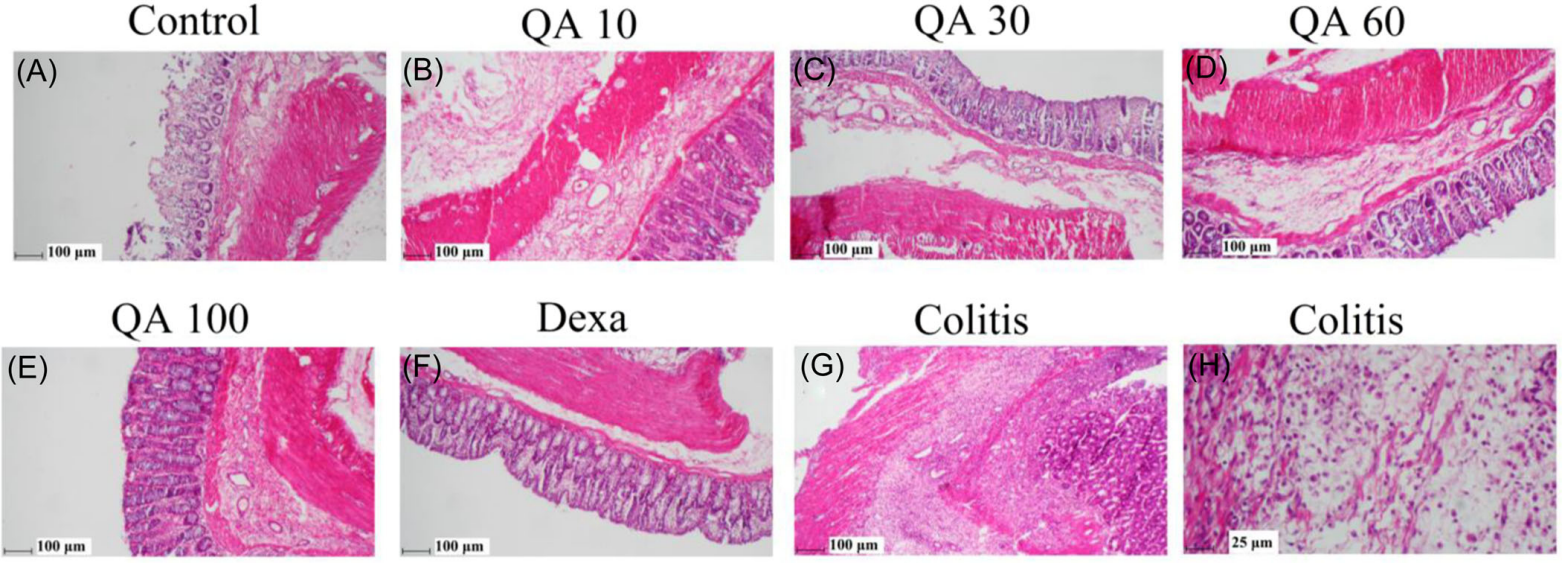
Microscopic changes of colon sections (staining with hematoxylin–eosin). QA 10, QA 30, QA 60, and QA 100: Quinic acid in dose of 10 (in 10× magnification and scale bar 100 μm), Quinic acid in dose of 30 (in 10× magnification and scale bar 100 μm), Quinic acid in dose of 60 (in 10× magnification and scale bar 100 μm), and Quinic acid in dose of 100 mg/kg (in 10× magnification and scale bar 100 μm); Dexa: Dexametazone (2 mg/kg) (in 10× magnification and scale bar 100 μm); g: Colitis: colitis group (in 10× magnification and scale bar 100 μm); h: Colitis: colitis group (in 40× magnification and scale bar 25 μm).

Figure 3 shows the microscopic images of colon tissues of various experimental groups after staining H&E. Figure 3A shows the normal microscopic appearance of the colon tissues in the control group. Severe damages were observed in the colon epithelium tissue of the colitis group (Figure 3G). In addition, severe ulcerations, necrosis, as well as cryptic abscess formations in the mucosa could be seen in this group. Furthermore, microscopic images clearly showed infiltration of inflammatory cells in all layers in the colitis group (Figure 3H).

Edema, lymphoid hyperplasia, lymphatic infiltration, and neutrophil infiltration were detected in all layers in QA‐treated groups in doses of 10 and 30 mg/kg, but on a smaller scale than the colitis group. The formation of Cryptic abscess and necrosis could be seen in the mucosa of these two groups. Ulcerations were also observed at 10 mg/kg in the QA group, but much milder than in the colitis group (Figure 3B). In the QA group at 30 mg/kg, ulceration was not found (Figure 3C).

QA groups in doses of 60 and 100 mg/kg showed much less damage to the epithelium. Edema, neutrophil infiltration, lymphoid hyperplasia, and infiltration in colon tissue were not significantly changed compared to other colitis groups. Necrosis and cryptic abscess formations were detectable in mucosa less than in other colitis groups, but no ulceration was found (Figure 3D,E).

Figure 3F shows the histopathological changes in the dexamethasone group. Edema, inflammation, cryptic abscess formations, ulceration, necrosis, neutrophil infiltration, and lymphatic infiltration were present in both submucosa and mucosa; however, less than in the colitis group.

Scoring related to histopathological changes in the studied groups is presented in Table 3. In all four cases, a significant difference could be seen in scoring between the colitis and control groups (*p* < .001).

**Table 3:** Scoring related to histopathological changes in the studied groups.

Scoring parameters	Histopathological scores						
	Control	Colitis	QA 10	QA 30	QA 60	QA 100	Dexa
Severity of inflammation	0.000	3.000 ± 0.2582###	2.833 ± 0.3073	2.500 ± 0.4282	1.500 ± 0.3416*	1.500 ± 0.3416*	1.667 ± 0.3333
Extent of inflammation	0.000	3.000 ± 0.2582###	2.500 ± 0.4282	2.500 ± 0.3416	2.333 ± 0.3333	2.333 ± 0.3333	2.333 ± 0.3333
Crypt damage	0.000	2.667 ± 0.4216###	1.667 ± 0.3333	1.667 ± 0.3333	1.500 ± 0.3416	1.333 ± 0.2108*	2.000 ± 0.2582
Involvement percent	0.000	3.500 ± 0.3416###	3.000 ± 0.2582	2.000 ± 0.2582**	2.000 ± 0.2582**	1.667 ± 0.2108***	1.833 ± 0.3073***

*Note*: Statistical analysis was performed using one‐way analysis of variance (ANOVA) test, followed by Tukey's post hoc test in GraphPad Software (version 8.4.3), sample number in each group = 8. Data are presented as mean ± SEM (*n* = 8).

Abbreviations: dexa, dexamethasone (2 mg/kg); QA 10, QA 30, QA 60, and QA 100, quinic acid in doses of 10, 30, 60, and 100 mg/kg.

^###^ *p* < .001 in comparison to the control values.

* *p* < .05 in comparison to the colitis values

** *p* < .01 in comparison to the colitis values

*** *p* < .001 in comparison to the colitis values.

Inflammation severity showed significantly different in the QA‐treated groups in doses of 60 (*p* < .05) and 100 mg/kg (*p* < .05) in comparison to the colitis values.

Inflammation extent was not statistically significant in either treatment group in comparison to the colitis values.

Regarding the crypt damage, there was a significant difference between the colitis group and QA group at 100 mg/kg (*p* < .05). Percentage of involvement was also different in the QA groups at 30 (*p* < .01), 60 (*p* < .01), 100 (*p* < .001) mg/kg, and dexamethasone at 2 mg/kg (*p* < .001) in comparison to the colitis values.

### 3.3 Effects of QA on SOD, CAT activity, and TAC level

**Figure 4:**
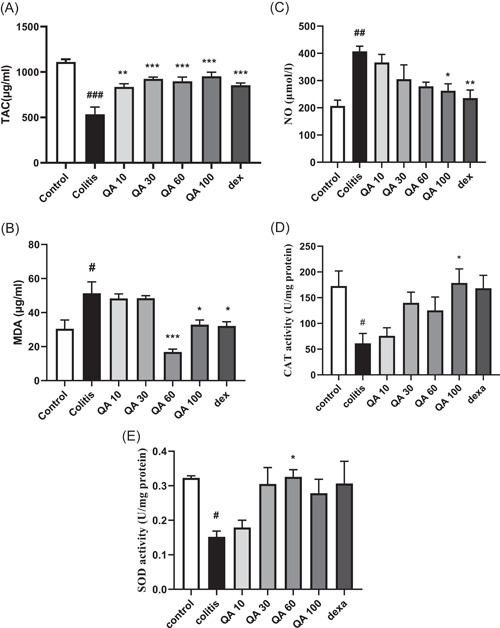
Changes in antioxidant and oxidative stress parameters in the tissue samples. Statistical analysis was performed using one‐way analysis of variance (ANOVA) test, followed by Tukey's post hoc test in GraphPad Software ersion 8.4.3), sample number in each group = 8. Data are presented as mean ± SEM (*n* = 8). #*p* < .05, ##*p* < .01, ###*p* < .001 in comparison to the control values. *p < .05, **p < .01, ***p < .001 in comparison to the colitis values. CAT, catalase; dexa, dexamethasone (2 mg/kg); MDA, malondialdehyde; NO, nitrite oxide; QA 10, QA 30, QA 60, and QA 100, quinic acid in doses of 10, 30, 60, and 100 mg/kg; SOD, superoxide dismutase; TAC, total antioxidant capacity.

As shown in Figure 4, colitis significantly reduced SOD, CAT activity (*p* < .05), and TAC level (*p* < .001), in comparison to the control values.

According to the results obtained, QA, in all four concentrations administrated, 10 (*p* < .01), 30 (*p* < .001), 60 (*p* < .001), 100 mg/kg (*p* < .001) respectively, and dexamethasone (*p* < .001), significantly increased the TAC level in comparison to the colitis values.

QA significantly increased SOD activity, at the concentration of 60 mg/kg (*p* < .05), and CAT activity, at the concentration of 100 mg/kg (*p* < .05) in comparison to the colitis values. The results related to SOD, CAT activity, and TAC level were presented both in the form of the graphic (Figure 4) and in the form of exact numbers in Table 4.

**Table 4:** Changes in antioxidant and oxidative stress parameters in the tissue samples.

Parameters	Groups						
	Control	Colitis	QA 10	QA 30	QA 60	QA 100	Dexa
TAC	1110 ± 30.40	534.0 ± 80.53###	835.8 ± 34.60**	924.9 ± 20.10***	897.7 ± 47.28***	953.7 ± 44.51***	853.9 ± 24.88***
NO	207.0 ± 20.98	407.3 ± 18.99##	366.5 ± 29.57	305.3 ± 52.50	278.8 ± 15.15	262.5 ± 25.47*	235.5 ± 29.87**
MDA	30.49 ± 5.148	51.33 ± 6.670#	48.29 ± 2.707	48.44 ± 1.430	16.82 ± 1.681***	32.87 ± 2.786*	32.12 ± 2.416*
CAT	172.7 ± 29.26	61.41 ± 19.15#	76.03 ± 15.52	140.0 ± 20.67	125.4 ± 25.98	178.7 ± 27.31*	168.4 ± 25.05
SOD	0.3228 ± 0.005916	0.1522 ± 0.01659#	0.1792 ± 0.02107	0.3051 ± 0.04766	0.3259 ± 0.02042*	0.2786 ± 0.04032	0.3063 ± 0.06443

*Note*: Statistical analysis was performed using one‐way analysis of variance (ANOVA) test, followed by Tukey's post hoc test in GraphPad Software (version 8.4.3), sample number in each group = 8. Data are presented as mean ± SEM (*n* = 8).

Abbreviations: CAT, catalase; dexa, dexamethasone (2 mg/kg); MDA, malondialdehyde; NO, nitrite oxide; QA 10, QA 30, QA 60, and QA 100, quinic acid in doses of 10, 30, 60, and 100 mg/kg; SOD, superoxide dismutase; TAC, total antioxidant capacity.

^#^ *p* < .05 in comparison to the control values.

^##^ *p* < .01 in comparison to the control values.

^###^ *p* < .001 in comparison to the control values.

* *p* < .05 in comparison to the colitis values

** *p* < .01 in comparison to the colitis values

*** *p* < .001 in comparison to the colitis values.

### 3.4 Effects of QA on NO and MDA level

In comparison to the control values, a significant increase was observed in the colitis values nitrite oxide (NO) (*p* < .01) and MDA (*p* < .05) levels.

QA, in 100 mg/kg concentration administrated, led to a significant reduction in both NO and MDA, in comparison to the colitis values (*p* < .05).

QA, in 60 mg/kg concentration administrated, led to a significant reduction in MDA, in comparison to the colitis values (*p* < .001). Dexamethasone had a similar effect in reducing both NO (*p* < .01) and MDA (*p* < .05).

The results related to NO and MDA levels were presented both in the form of the graphic (Figure 4) and in the form of exact numbers in Table 4.

### 3.5 Effects of QA on inflammatory gene expressions

**Figure 5:**
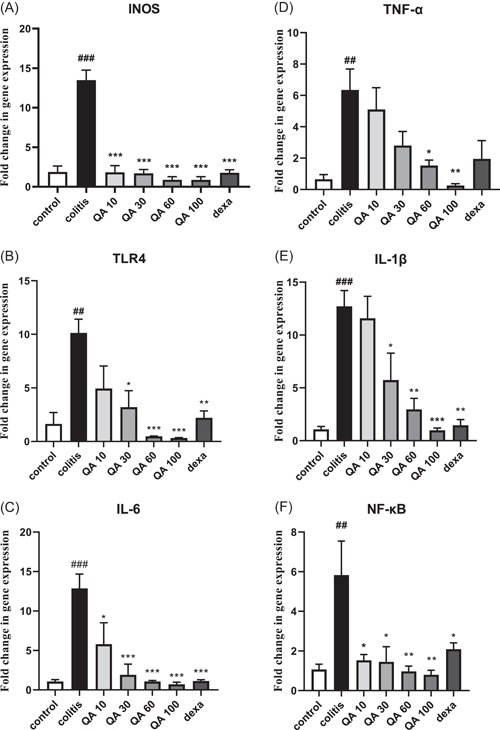
Genes expression related to colon tissue inflammation. Statistical analysis was performed using one‐way analysis of variance (ANOVA) test, followed by Tukey's post hoc test in GraphPad Software (version 8.4.3), sample number in each group = 8. Data are presented as mean ± SEM (*n* = 8). #*p* < .05, ##*p* < .01, ###*p* < .001 in comparison to the control values. *p < .05, **p < .01, ***p < .001 in comparison to the colitis values. dexa, dexamethasone (2 mg/kg); QA 10, QA 30, QA 60, and QA 100, quinic acid in doses of 10, 30, 60, and 100 mg/kg.

According to presented findings in Figure 5, the inflammatory gene expressions were significantly elevated in colitis rats, in comparison to the control values, as follows,

INOS (*p* < .001, F.C: 7.213), TLR4 (*p* < .01, F.C: 6.17), IL‐6 (*p* < .001, F.C: 12.24), TNF‐α (*p* < .01, F.C: 9.62), IL‐1β (*p* < .001, F.C: 11.86), and NF‐κB (*p* < .01, F.C: 5.5) (F.C: fold change).

QA administration in 10 (*p* < .001, F.C: −7.40), 30 (*p* < .001, F.C: −7.40), 60 (*p* < .001, F.C: −15.38), and 100 mg/kg (*p* < .001, F.C: −15.62) concentrations, as well as dexamethasone (*p* < .001, F.C: −7.6), significantly decreased INOS expression in comparison to the colitis values (Figure 5A).

QA changed other genes as follows: 30 (*p* < .05, F.C: −3.22), 60 (*p* < .001, F.C: −20.8), and 100 (*p* < .001, F.C: −32.25) mg/kg also dexamethasone (*p* < .01, F.C: −4.6), significantly attenuated TLR4 expression, in comparison to the colitis values (Figure 5B).

Dexamethasone (*p* < .001, F.C: −11.49) and QA at 10 (*p* < .05, F.C: −2.12), 30 (*p* < .001, F.C: −6.75), 60 (*p* < .001, F.C: −12.19), and 100 (*p* < .001, F.C: −18.51) mg/kg attenuated the IL‐6 expression in comparison to the colitis values (Figure 5C).

QA at 60 (*p* < .05, F.C: −4.1) and 100 (*p* < .01, F.C: −25.64) mg/kg also attenuated the TNF‐α gene expression in comparison to the colitis values (Figure 5D).

Dexamethasone (*p* < .01, F.C: −9.09) and QA at 30 (*p* < .05, F.C: −2.2), 60 (*p* < .01, F.C: −4.3), and 100 (*p* < .001, F.C: −12.98) mg/kg attenuated the IL‐1β expression in comparison to the colitis values (Figure 5E).

Finally, dexamethasone (*p* < .05, F.C: −2.80) and QA at 10 (*p* < .05, F.C: −3.84), 30 (*p* < .05, F.C: −4.16), 60 (*p* < .01, F.C: −6.25), and 100 (*p* < .01, F.C: −7.29) mg/kg, attenuated NF‐κB gene expression in comparison to the colitis values (Figure 5F).

### 3.6 Effects of QA on apoptotic gene expressions

**Figure 6:**
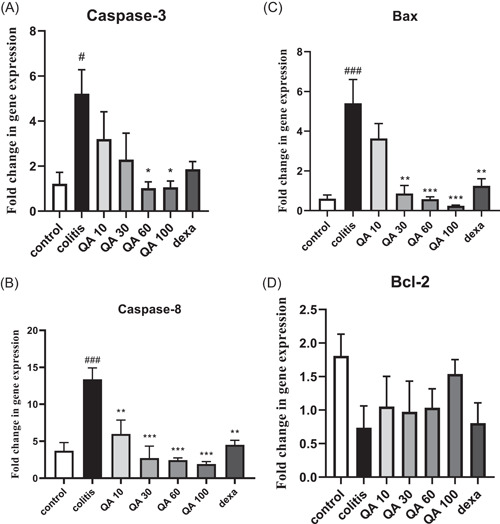
Genes expression related to colon tissue apoptosis. Statistical analysis was performed using one‐way analysis of variance (ANOVA) test, followed by Tukey's post hoc test in GraphPad Software (version 8.4.3), sample number in each group = 8. Data are presented as mean ± SEM (*n* = 8). #*p* < .05, ##*p* < .01, ###*p* < .001 in comparison to the control values. *p < .05, **p < .01, ***p < .001 in comparison to the colitis values. dexa: dexamethasone (2 mg/kg); QA 10, QA 30, QA 60, and QA 100: quinic acid in doses of 10, 30, 60, and 100 mg/kg.

Apoptotic gene expressions, including Bax (*p* < .001, F.C: 9.15), Caspase‐3 (*p* < .05, F.C: 4.30), and Caspase‐8 (*p* < .001, F.C: 3.61) were significantly enhanced in the colitis group compared to the control values (Figure 6).

Based on the results (Figure 6A), QA in concentration of 60 (*p* < .05, F.C: −5.10) and 100 (*p* < .05, F.C: −4.95) mg/kg decreased the Caspase‐3 expression and at 10 (*p* < .01, F.C: −2.27), 30 (*p* < .001, F.C: −5), 60 (*p* < .001, F.C: −5.55), and 100 (*p* < .001, F.C: −7.14) mg/kg decreased Caspase‐8 expression, in comparison to the colitis values. Also, dexamethasone attenuated Caspase‐8 expression (*p* < .01, F.C: −3.03; Figure 6B).

Dexamethasone (*p* < .01, F.C: −4.54) and QA at 30 (*p* < .01, F.C: −6.25), 60 (*p* < .001, F.C: −10), and 100 (*p* < .001, F.C: −22.72) mg/kg decreased the Bax expression in comparison to colitis values (Figure 6C).

The change in Bcl‐2 gene expression was not statistically significant in all groups (Figure 6D).

## 4 DISCUSSION

In this study, it was shown that the level of TAC and cell defense mechanisms, including antioxidant enzymes (SOD and CAT) was reduced, while oxidative stress indices such as MDA and NO were significantly increased, following induction of colitis.

Colitis caused a significant increase in the expression of inflammatory and apoptotic genes, such as IL‐6, IL‐1β, TNF‐α, TLR4, INOS, NF‐κB, Bax, Bcl‐2, Caspase‐3, and Caspase‐8, that led to severe inflammation in the colitis rats, as well as infiltration of inflammatory cells and severe damage to colon tissue epithelium. Results provided in therapeutic groups revealed that QA was able to improve inflammation complications, apoptosis, and histopathological injuries, as well as oxidative stress conditions, caused by colitis.

Inflammation and oxidative stress are among the complications of UC disease playing major roles in disease pathogenesis.^27^, ^28^, ^29^, ^30^ Due to the increased secretion of reactive oxygen metabolites and pro‐inflammatory cytokines from macrophages, the inflammatory mediators, such as TNF‐α, IL‐1β, and IL‐6, in the colon tissue of patients with UC, increase.^29^, ^31^

The interactions of TLR4 and LPS (lipo poly saccharide) in bacterial cell walls lead to the activation of TLR4 pathway. TLR4 pathway is an important signaling pathway, which is activated during damage and inflammation.^32^ Activation of this pathway causes the activation of a transcription factor, present in the cytoplasm (NF‐κB).^31^ NF‐κB after translocating into the nucleus, activates transcription of IL‐6, IL‐1β, and TNF‐α genes.^33^ Previous studies have shown that NF‐κB upregulation, stimulates the expression of INOS, and consequently NO production.^32^, ^34^ NO is a potent pro‐inflammatory mediator that studies have reported its increased levels in the inflamed tissue of the colon. It has an important role in the production of inflammatory cytokines in relevant areas.^35^ On the other hand, damage to the colon, increases due to increased levels of NO and, following that, reactive nitrogenous species, and reactive oxygen species.^36^

It indicates the importance of NF‐κB‐INOS‐NO signaling pathways in imposing oxidative stress and inflammation due to colitis.^34^, ^37^ So, the secretion of reactive products, including peroxides and superoxides, in inflamed mucosa, from active leukocytes, increases, and exacerbates oxidative damages to colon tissue.^28^, ^38^, ^39^ When superoxides are converted to hydroxyl radicals, interact with the fatty acids and increase the MDA production and decrease the activities of antioxidant enzymes, including SOD and CAT. Previous studies have shown that both enzymes are reduced in colitis.^40^ In the present study, according to the results of previous studies, the expression of inflammatory cytokines (TLR4, TNF‐α, NF‐κB, INOS, IL‐6, and IL‐1β) and oxidative stress indicators (NO and MDA) were increased and, the levels of TAC and antioxidant enzymes (CAT and SOD) were significantly decreased, due to colitis induction.

In active colitis, due to apoptosis and acceleration of epithelial cell turnover, an epithelial cell depletion occurs, leading to an increase in intestinal permeability and pathogenic microorganism invasion.^41^ Previous studies have shown that, Caspase‐3, Caspase‐8, Bax, and Bcl‐2 are among the most important cytokines in the process of apoptosis.^42^ In pathological conditions such as colitis, first procaspase‐8 is activated, and subsequently, the apoptotic cascade begins, which further leads to the activation of procaspase‐3.^43^ This process eventually leads to DNA fragmentation and cell death.^44^ On the other hand, activation of caspases causes migration of neutrophils and activation of NF‐ҡB, which induces ROS production and eventually the mucosal lesion.^32^, ^43^

NF‐ҡB is also involved in the activation of apoptotic regulatory genes.^43^ The increased Bax expression and the decreased Bcl‐2 expression are associated with increased apoptosis in the colon tissue epithelial cells.^44^ As the results of our study, in confirmation of previous studies showed, the induction of colitis increased the expression of apoptotic genes, including Caspase‐3, Caspase‐8, and Bax. Previous studies have shown that inflammation, epithelial damage, edema, and in acute cases, necrosis and ulceration are among injuries to the colon tissue, due to the administration of acetic acid.^8^, ^45^, ^46^ Histopathological results from the other studies have also reported severe damage to the epithelium, cryptic abscess formations, hyperplasia, and hyperemia in colon tissue due to the induction of colitis.^47^, ^48^ It has also been suggested that free radicals are involved in the morphological changes and epithelial damage to colon tissues.^49^ Studies have confirmed that, caspase activations increase the level of apoptosis in the epithelial cells, and consequently intensify epithelial damage.^32^, ^50^ In the present study, according to the results of previous studies, colitis led to the infiltration of inflammatory cells, epithelium damage, and edema. The common medications used for colitis treatment, in different people, produce a variety of side effects and responses, as well as short‐term treatment results. Herbal therapy has received a lot of attention due to the good results and fewer side effects.^51^ QA is a phenolic acid compound with the structure of cyclohexane carboxylic acid.^13^ This natural compound is found in the bark of the cinchona, coffee beans,^13^ and other plants, including sweet potatoes, apples, and peaches.^52^ Previous studies have confirmed the antioxidant and anti‐inflammatory properties of QA.^16^ It should be noted that the most of the existing studies have been performed on QA conjugates, such as chlorogenic acid, which is an ester of caffeine and QA. When chlorogenic acid reaches the large intestine, it is hydrolyzed by microbial esterases to release caffeine and QA.^53^ A study conducted by Gao et al., confirmed the positive chlorogenic acid effects on DSS‐induced UC in mice. In this study, chlorogenic acid decreased inflammatory factors, and increased anti‐inflammatory factors,^54^ also reduced colonic mucosal damage and inflammation of the colon, as well as inhibited oxidative stress, and apoptosis.^54^ In a study conducted by Shi et al., *Schisandra chinensis*, which contains QA, significantly increased GSH level and SOD activity and decreased myocardial MDA.^55^ It also inhibited H_2_O_2_‐induced apoptosis by downregulating Caspase‐3, Bax, and cytochrome‐C expression and upregulating the Bcl‐2 mRNA expression.^55^

Plants metabolites, including flavonoids, terpenes, and polyphenols are natural compounds with significant effects in improving colitis, due to their antioxidant and anti‐inflammatory properties.^8^, ^56^, ^57^ Power of the free radical scavenging is one of the proposed mechanisms for their therapeutic actions,^8^ which reduces MDA production, by inhibiting lipid peroxidation, and preventing the reduction of antioxidant enzymes.^9^ Flavonoids and polyphenols inhibit NF‐κB signaling in the colon tissue, through interaction with TLR4.^58^, ^59^ As a result, the expression of TNF‐α, IL‐1β, and IL‐6, as well as the production of inflammatory cytokines are reduced. In addition, inhibition of NF‐κB is accompanied by inactivation of INOS and a decrease in NO level.^60^ The results of the present study also showed QA significantly reduced the expression of inflammatory cytokines (IL‐6, TLR4, IL‐1β, INOS, TNF‐α, and NF‐κB), as well as MDA and NO, in contrast increased TAC, SOD, and CAT activity, significantly. Therefore, the antioxidant and anti‐inflammatory properties of QA can be considered relevant to inhibition of the TLR4‐NF‐κB signaling cascade and NF‐κB‐INOS‐NO signaling pathway, also the power of free radical scavenging of QA. Furthermore, the positive effects of QA in improving histological damage could be related to the antioxidant activity of this compound, and also, the ability to inhibit apoptosis.

Study limitations were as follows, animal experiments are time‐consuming and expensive. Besides that, according to the Ethical Guidelines for the Use of Animals in Research, for minimizing the number of animals in experiments, it was not possible to check more doses of the drug on more animals, and it was also not possible to perform pretreatment or posttreatment tests.

Also to reduce animal suffering, it was not possible to carry out experiments for more days. It is suggested that such experiments be performed as separate studies, in completing the present study.

## 5 CONCLUSION

QA ameliorated UC through the inhibition of two TLR4‐NF‐κB and NF‐κB‐INOS‐NO signaling pathways, which results in the reduction of colitis complications, including oxidative stress, inflammation, apoptosis, and histopathological injuries in rats. Therefore it can be concluded, that QA exerts its therapeutic effects through antiapoptotic, antioxidant, and anti‐inflammatory properties.

## AUTHOR CONTRIBUTIONS

**Maryam Ghasemi‐Dehnoo**: Data curation; formal analysis; investigation; writing—original draft; writing—review and editing. **Zahra Lorigooini**: Conceptualization; investigation; project administration; writing—review and editing. **Hossein Amini‐Khoei**: Conceptualization; investigation; project administration; writing—review and editing. **Milad Sabzevary‐Ghahfarokhi**: Data curation; formal analysis; investigation. **Mahmoud Rafieian‐Kopaei**: Conceptualization; investigation; project administration; supervision; writing—review and editing.

## CONFLICT OF INTEREST STATEMENT

The authors declare no conflict of interest.

## Data Availability

Data regarding the present study are available at Medical Plants Research Center, Shahrekord University of Medical Sciences.
